# Polyarthrite rhumatoïde vieillie avec poumon rhumatoïde: une observation caricaturale

**DOI:** 10.11604/pamj.2018.30.253.16372

**Published:** 2018-08-06

**Authors:** Ines Kechaou, Imène Boukhris

**Affiliations:** 1 Service de Médecine Interne B, Hôpital Charles Nicolle, Université de Tunis El Manar, Faculté de Médecine de Tunis, Tunis, Tunisie

**Keywords:** Polyarthrite rhumatoïde, sujet âgé, pneumopathie infiltrante diffuse, Rheumatoid arthritis, elderly, diffuse infiltrative pneumopathy

## Abstract

In the elderly, rheumatoid arthritis is defined by an average age of onset over 60 years. Prognosis mainly depends upon functional capacity, evolutionary stage of the disease, onset or no onset of joint malformations. We report the case of a 70-year old female patient with a history of arterial hypertension, hypothyroidism and Sjögren syndrome diagnosed in 2005. The patient was lost to follow-up and hospitalized in our department in 2016 for diffuse polyarthralgies of large joints with biologic inflammatory syndrome. Clinical examination showed cachectic patient (BMI: 17,6Kg/m^2^). She had articular deformations: hand arthritis with a “camel back” appearance and a “cubital gust of wind” appearance (A), claw feet and bilateral progression of hallux valgus (B). Lung auscultation showed diffuse crackling sound in both lungs. The patient didn't have dyspnea. Cardiac auscultation was normal and there were no signs of heart failure. Chest x-ray showed diffuse reticulo-micronodular appearance (C). Chest CT scan showed diffuse infiltrating pneumopathy supporting common interstitial lung disease (ILD) (D). Doppler echocardiography showed moderate pulmonary arterial hypertension 40mmHg. Blood gas test showed hypoxia 81mmHg with a saturation of 96%. The diagnosis of senile rheumatoid arthritis complicated by pulmonary involvement was then retained. Functional prognosis was uncertain due to these very severe joint deformations.

## Image en médecine

La polyarthrite rhumatoïde du sujet âgé se définit par un âge de début supérieur à 60 ans. Le pronostic est surtout fonctionnel, dépendant du stade évolutif de la maladie, de l'installation ou pas de déformations articulaires. Nous rapportons l'observation d'une patiente âgée de 70 ans aux antécédents d'hypertension artérielle, d'hypothyroïdie et de syndrome de Sjögren diagnostiqué en 2005. Elle a été perdue de vue et hospitalisée dans notre service en 2016 pour polyarthralgies diffuses des grosses articulations avec syndrome inflammatoire biologique. A l'examen, la patiente était cachectique (BMI: 17,6Kg/m^2^). Elle avait des déformations articulaires: main rhumatoïde avec un aspect en dos de chameau et de coup de vent cubital (A) et au niveau des pieds, des orteils en griffe et un hallux valgus évolué bilatéral (B). L'auscultation pulmonaire a révélé la présence de râles crépitant diffus aux deux champs pulmonaires. La patiente n'était pas dyspnéique. L'auscultation cardiaque était normale et il n'y avait pas de signes d'insuffisance cardiaque. La radio thorax avait montré un aspect réticulo-micro-nodulaire diffus (C) avec au scanner thoracique un aspect de pneumopathie infiltrante diffuse compatible avec une pneumopathie interstitielle usuelle (UIP) (D). L'échographie cardiaque avec doppler avait montré une hypertension artérielle pulmonaire modérée à 40mmHg. Les gaz du sang ont révélé une hypoxie à 81mmHg avec une saturation à 96%. Le diagnostic de polyarthrite rhumatoïde vieillie compliquée d'une atteinte pulmonaire a été alors retenu. Cette patiente présente un pronostic fonctionnel réservé devant ces déformations articulaires très importantes.

**Figure 1:**
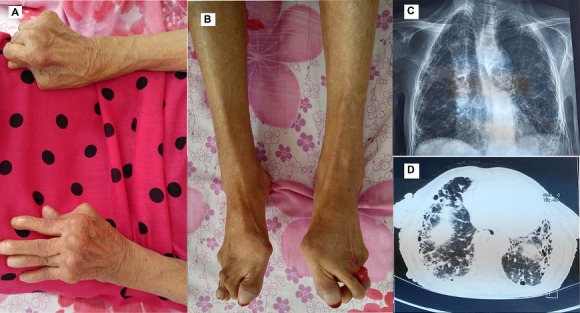
A) déformations évoluées des mains avec un aspect en dos de chameau et doigts en col de cygne de la main gauche, ankylose en flexion des doigts de la main droite; B) hallux valgus évolué des pieds; C) radiographie du thorax montrant des opacités réticulo-micro-nodulaires pulmonaire; D) scanner thoracique montrant un aspect de fibrose diffuse

